# Distribution pattern of Crimean–Congo Hemorrhagic Fever in Asia and the Middle East

**DOI:** 10.3389/fpubh.2023.1093817

**Published:** 2023-01-26

**Authors:** Munazza Aslam, Rao Zahid Abbas, Abdullah Alsayeqh

**Affiliations:** ^1^Department of Pathology, Faculty of Veterinary Science, University of Agriculture, Faisalabad, Pakistan; ^2^Department of Parasitology, Faculty of Veterinary Science, University of Agriculture, Faisalabad, Pakistan; ^3^Department of Veterinary Medicine, College of Agriculture and Veterinary Medicine, Qassim University, Buraidah, Qassim, Saudi Arabia

**Keywords:** CCHF, prevalence, geographical distribution, pathophysiology, risk factors, zoonosis, ribavirin, prevention

## Abstract

Crimean–Congo Hemorrhagic Fever (CCHF) is one of the most important vector-borne diseases of zoonotic potential that can be acquired following the bite of the *Hyalomma* species of ticks. It is a highly prevalent disease in Asia and the Middle East. The risk factors of this disease are contact with infected tissue, blood, patient, or livestock in the acute viremic phase, infected tick bites, or the manual removal of ticks. The disease is clinically described as progressive hemorrhages, fever, and pain in musculature. Biochemical tests reveal elevated levels of creatinine phosphokinase, alanine transaminase, aspartate aminotransferase, and lactate dehydrogenase. Clotting time is prolonged in pro-thrombin tests, and pathogenesis is mostly related to the disruption of the epithelium during viral replication and indirectly by secreting cytotoxic molecules. These molecules cause endothelial activation and result in the loss of function. Supportive therapy is given through blood or plasma infusions to treat or manage the patients. According to the most advanced studies, CCHF can be treated by Ribavirin, which is an antiviral drug that shows excellent results in preventing the disease. Health-care staff are more prone to infection. The hemorrhagic phase represents a high risk for accidental exposures. This literature review presents a comprehensive overview of the viral epidemiology, zoonotic perspectives, and significant risk factors of CCHF in various Middle East and Asian countries. Furthermore, the pathophysiology and preventive strategies of CCHF have also been discussed as well as legislation and policies regarding public outreach programs, research, and development aimed at infection prevention and control that are required at a global level.

## 1. Introduction

**Figure 1:**
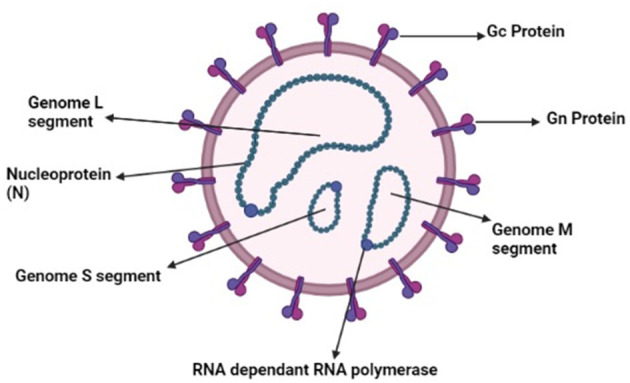
Schematic diagram of Nairo virus (Drawn by BioRender app).

Ticks and tick-borne diseases have been a threat to humans and animals for many years (1–4). Ticks play an important role as a vector for the transmission of several diseases (5–9). Tick-borne viruses belong to the *Bunyavirales* and *Mononegavirales* orders. These orders contain nine families that cause tick-borne diseases (10). Southeast Asian countries are more vulnerable because of the increasing population and the developing nature of healthcare infrastructure and communities. Crimean-Congo Hemorrhagic Fever (CCHF) is a life-threatening zoonotic disease that affects a vast geographical area (11). CCHF is caused by a virus that belongs to the genus *Nairo* virus, and its family is *Nairoviridae*. It is a negative sense RNA virus containing a segmented genome that is further divided into small (S), medium (M), and large (L) segments as illustrated in Figure 1. The small segment is responsible for the diversity among the viral isolates of different regions (12). The *Hyalomma* tick is responsible for its spread to animals and humans by salivary pathways. CCHF is virulent and potentially hazardous, with the ability to be used as a bioterrorism weapon. CCHF causes 3 to 30% mortality in humans, and becomes disastrous when occurs above the endemic level (13). Tick-bites, animals in the viremic phase, and contact with the blood of an infected patient in the acute phase of infection are all sources of transmission of infection (14). Clinically, the disease is characterized by fever, extensive hemorrhages, and myalgia. Some signs and symptoms, such as hepatomegaly and splenomegaly, are mostly observed in different regions where the disease is prevalent, and these typical signs also vary according to the geographical area and the types of vectors (15). Ribavirin is used as a treatment for CCHF. Effectiveness of the medicine is based only on observational studies, thus it is tentative (16). A few studies suggest that certain beneficial effects are associated with the use of ribavirin (17). In high-risk exposures, treatment is done *via* the use of ribavirin (16). However, there is controversy over the effectiveness of ribavirin as a treatment of CCHF (18).

The fatality rate of the disease warrants the adoption of preventive measures against the CCHF virus (19). In geographical areas where the disease is endemic due to the abundance of tick vectors, one should use protective clothing when these ticks are biologically active and are transmitting the virus to humans (20). The use of repellents and examining the skin and clothing for the removal of ticks could spare tick bite incidents (21). People who live in urban areas are at higher risk of exposing themselves to viremic animals, and, therefore, are advised to wear gloves while handling animal tissue or blood to avoid infection (19). The use of protective aprons or clothing is necessary while treating viremic animal herds. Medical staff around CCHF-infected patients are advised to keep barriers while providing medical care (22). The use of goggles, face shields, gowns, and gloves is mandatory when treating patients or soiled surfaces (23). Health-care staff who experience needle stick injury are administered ribavirin by parenteral route to spare the chances of contracting the viral infection (24).

Further research studies are required to reveal risk factors and transmission patterns of the virus among various hosts (25). The biological roles of previously described vectors need to be discussed in detail while risk factors, such asclimatic changes, reservoir hosts, and other contributory factors, need more investigation. This sort of work requires a consortium that takes on board multidisciplinary professionals for infection prevention and control. Epidemiologists, microbiologists, entomologists, and veterinarians should work together under the theme of One Health to devise ways to curtail the occurrence of the disease. There must be rapid risk communication between these disciplines and ecologists in order to avoid the disease in a particular geographical area. Certain drug trials are needed to develop a drug of choice for CCHF, e.g., heparin and other anti-coagulants can be tested to treat disseminated intravascular coagulation. The prevalence of the CCHF virus in Asian and Middle Eastern countries, its association with humans, pathogenesis, pathophysiology, and treatment strategies of the disease have been discussed in the following review.

## 2. Viral clades

The genomic clades of CCHF are Clade I, which involves the region of West Africa, Clade II has been found in Central Africa, while Clade III has been found in South and West Africa. Clade IV exists in the Middle East and Asia, while Clade V and VI belong to Europe and Greece, respectively. The asian Clade is further divided into two distinct clades, Asia 1 and Asia 2, respectively (12). This phylogenetic classification of the virus is done based on S segments of the CCHF virus (26).

## 3. Pattern of distribution in Asian and Middle Eastern countries

**Figure 2:**
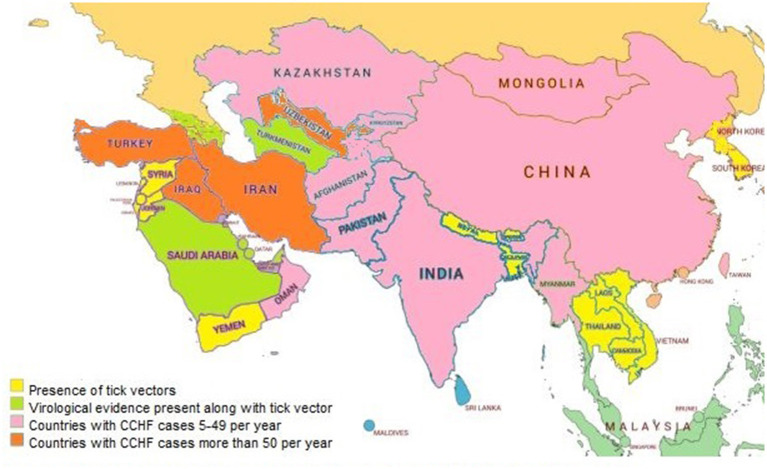
Distribution pattern of CCHF in Asian and Middle East countries.

CCHF is a highly prevalent disease involving different countries of Asia and other continents (27). The prevalence of CCHF in Asian and Middle Eastern countries is described country-wise in Figure 2.

### 3.1. Pakistan

CCHF cases in the country are increasing with each passing year (28). In the 1960's, the virus was first identified in ticks infesting local livestock (29). In 1976, CCHF was observed in Pakistan for the first time as a human case. Up to 2010, only 14 cases were reported (30). After 2010, CCHF cases began to increase at a rapid rate. From 2014 to 2020, more than 350 cases of CCHF were confirmed by the National Institute of Health, Islamabad (31). The mortality rate was proposed to be more than 25% (32). Among these cases of CCHF, only 38% were reported from the Balochistan province, 23% were from the Punjab province, 19% from Khyber Pakhtunkhwa, 14% from Sindh, and 6% were reported from the capital city Islamabad (33). In another research study, the prevalence of CCHF was recorded to be 24.7% in Punjab, 16.2% in Sindh, 52.4% in Khyber-Pakhtunkhwa, and 59.3% in Baluchistan (34). The disease was more prevalent in rural areas due to the close interaction of people with the animals. People who live in urban areas tend to be more infected and at a higher rate on the eve of *Eid Ul Adha*, when sacrificial animals are sold and then consumed without proper inspection of animal/animal by-products (35).

Prevalent strains from neighboring countries like Iran and India tend to spread and circulate in Pakistan, and vice versa (36). In 2004, 248 cases were positive for CCHF and, among those cases, only 68% were reported from Baluchistan (Pakistan) and Sistan of Iran (37). In the period from 2004 to 2006, there were annual increases in cases of up to 300 patients. On average, 6% of ticks were positive for harboring the virus, and the Asia 1 and Asia 2 were prevalent strains (38). They are considered clade IV among the genomic sequence.

There are certain risk factors that are playing a crucial role in the spread of CCHF. Rapid climatic changes result in biannual peaks between March to May and August to October. Poor sanitation, unhygienic slaughterhouses, transport of animals within cities, nomadic lifestyle, and lack of trained animals and medical care staff contribute to the spread of CCHF (39).

### 3.2. China

In 1965, there were reports of hemorrhagic fever in western China. The samples collected from ticks, animals, and humans yielded the CCHF virus upon diagnosis. From 1965 to 1994, 260 farmers were reported to be infected with CCHF. The mortality rate was 80% (40). One imported case was reported in 2013 in the city of Beijing, China. Confirmed reports of the CCHF virus were also received from Xinjiang, Yunnan, and Qinghai provinces in China (41, 42). The CCHF virus was detected among ticks of sheep and camels in the Inner Mongolia region of China (43). From 1951 to 2021, only 447,848 cases were reported to be infected from *bunyavirales* viruses, and CCHFV, along with three other viruses, was reported to cause the most disease burden (44).

### 3.3. Kazakhstan

In 1944, CCHF was reported for the first time in Kazakhstan (45). Most of the prevalence was reported in Zhambyl and Kyzylorda, regions of the south of Kazakhstan (46). The first reported case of CCHF was in the Turkestan region and was termed Central Asian Fever (47). Later, in 1963 and 1982, cases were reported from Kyzylorda (48) and Zhambyl (49). These regions show a high prevalence of CCHF every year. The mortality rate from CCHF was 14.8% (50) and about 16 cases were reported annually in Kazakhstan (51). In the Zhambyl region, cattle and sheep were tested for the virus and the CCHF virus was found in ~2.4% of sheep and 3.8% of the cattle population (52).

### 3.4. India

The first case of CCHF was identified in the Gujrat state of India, and it was the result of a nosocomial infection that was related to Pakistan across the border (53). A local survey of livestock revealed that serum and tissue were analyzed to check the prevalence of *H. anatolicum* (54). From 2010 to 2019, 34 outbreaks were reported from the region of Uttar Pradesh, Rajasthan, and Gujarat (55). Eight secondary cases of CCHF were reported out of the 34 cases (56). In another study, CCHF cases were detected in four states of India (57).

### 3.5. Afghanistan

In 2009, there was an outbreak of CCHF in the region of Herat, Afghanistan. Only 60 positive cases were detected. It was revealed that native breeds of cattle and sheep were harboring high levels of IgG in their blood in the surrounding area, indicative of probable pre-exposure (58). In a study, 51 positive cases of CCHF were detected by ELISA and, of these, 11 patients died. These were butchers and shepherds. Patients with CCHF increased significantly between June to September of the endemic year. This proved that lifestyle and climatic conditions are the risk factors for the spread of the disease (59). Vector ticks of CCHF were identified on the border of Afghanistan and Iran, giving rise to the potential risk of CCHF in humans (60).

### 3.6. Malaysia

In a research study, the seroprevalence of CCHF was determined in 2015 within the Orang Asli, a minority population. The titer was too low in them for detection, and it was negative in other populations (61).

### 3.7. Iran

In the 1970's, the first case of CCHF was reported in Iran, based on the presence of antibodies against CCHF in the serum of cattle, sheep, and humans (62). In Tehran, a sheep abattoir was found to be harboring viral antigens (63). The source of the virus was the ticks of the *Ixodes* genus (64). Human disease reports were reported in 1999 and since then CCHF outbreaks are reported from different regions of the country (65). The mortality rate was 20% in 2000 and it reduced to 6% in 2007 (66). In a study, 203 ticks were checked for the presence of CCHFV and this was absent in the Kerman province although it was an endemic region (67). In 2011, seven butchers from 104 slaughterhouses were detected to be seropositive for CCHF (68).

### 3.8. United Arab Emirates

In 1979, CCHF was reported for the first time in Dubai, in the Arabian Peninsula, in a health facility outbreak (69) and, thereafter, no other case was observed until 1994 when an epidemic was reported in the United Arab Emirates among abattoir workers (70). A serum investigation was done in native and imported breeds of livestock to determine the antibody titer, and results showed that native breeds were positive for CCHF antibodies while the imported breeds lacked the CCHF antibody titer. This further confirmed the presence of the disease in that region. Livestock import was reported to be the major cause of the disease (71). It also led to 35 confirmed cases of CCHF with clinical pictures (70). In 2010, two cases of CCHF were reported in Dubai (72). Five cases and two deaths were reported between the time frame from 1998 to 2013 (73). *Hyalomma* tick presence was proven in native livestock giving rise to an alarming situation for the risk of spread of CCHF (74, 75).

### 3.9. Oman

In the mid-1990's, human-infected cases of CCHF were reported from the region of Oman, and the serum analysis of local animals confirmed the presence of the CCHF virus in the region (76). The first case of CCHF was observed in 2011, which was the first case after 15 years (77). In 2014, one death, along with a further 18 human-infected cases, were reported in Oman. Only 16 confirmed cases of CCHF were reported in 2015. The mode of disease transmission was either slaughtered animals or livestock (78). CCHF was transferred *via* occupation in most of the cases occurring in the period from 2011 to 2017 (79). In 2019, from the Northern region of Oman, four patients with CCHF were reported during the festival of *Eid Ul Adha* (80).

### 3.10. Iraq

Due to war and civil unrest, data collection for CCHF was not efficiently done in Iraq, but certain reports claimed the presence of CCHF in the country (81). Six cases of CCHF were reported between 1989 and 2009. In 2010, 11 cases of CCHF were reported. Three fatal cases were reported in 2018, while 33 cases were reported in 2021, with 13 fatal cases (82). The World Health Organization (WHO) reported 1085 suspected cases of CCHF in 2022, and laboratory confirmation revealed 287 positive cases, with 83 deaths being of suspected CCHF patients and 52 patients confirmed patients of CCHF (18.1% case fatality rate) (83).

### 3.11. Kuwait

From 1979 to 1982, a serological analysis of 502 patients was done to confirm the disease in two hospitals. Only 18 cases were reported to harbor the disease. According to a research study, only 17 patients who had a close association with livestock had pathognomonic signs of CCHF. About 38% of patients were from rural backgrounds. They were located on the borders of Kuwait so there were chances of imported cases of CCHF (84).

### 3.12. Egypt

Egypt is a transcontinental country and it showed infections of CCHF in 1978 in many wild and domestic animals. According to a serological study, camels had 8.8% titer while sheep demonstrated 23.1% titer (85). From 1986 to 1987, camel import was the reason for the spread of the disease (86). Antibodies against the CCHF virus were detected again in a 2004 to 2005 serological survey, which confirmed the role of ruminants as a maintenance host (87). Human cases were also observed, mostly in health-care workers and people of rural backgrounds. In 1981 and 2012, a total of four cases were detected, with one death (88, 89).

### 3.13. Saudi Arabia

In 1990, a case of CCHF was first reported in the country. According to this report, seven people were infected with the virus in Makkah (90). From 1989 to 1990, only 40 workers of slaughterhouses showed suspected signs of CCHF in Makkah, and twelve patients died due to these suspected signs. Imported sheep were considered to be the source of the disease (91). Imported animals and humans who were working on the seaport were also tested for titer of antibodies. Animals along with the staff of the seaport of Jeddah also tested positive for the presence of antibodies against CCHF (92).

### 3.14. Turkey

It has been observed that Turkey is the hub of CCHF, with reports of about 1,000 confirmed cases per year. In the past few decades, the country was CCHF-free, but with time, Turkey became the lodestone of the disease. It may be possible that there was underreporting or that CCHF was not differentiated from other diseases or was misdiagnosed as some other disease, but it might have been present in the region. Moreover, the ecological and environmental conditions in the country are very favorable for the successful completion of tick life cycles (93). In 2002, the first case of CCHF was reported in patients in the region of the eastern Black Sea in Turkey (93). In 2009, the fatality rate among 500 patients was reported to be 5% (94). This rate was high because initially the disease was misdiagnosed in 66% of patients, as the early symptoms of the disease were not pathognomonic (95). According to a serosurvey conducted in the region of eastern Turkey and Anatolia, specific antibodies against the CCHF virus were about 80%, indicating that the region was most prone to the disease among all regions of Turkey (96). *Hyalomma* ticks were used for viral isolation, and about 20% of them harbored the virus (97). In a study, conducted at a secondary care hospital in Kastamonu, patients with CCHF were evaluated in the period of 2014–2017. A total of 76 suspected cases appeared, and CCHF was confirmed in 46.1% of cases. During those 4 years, the case fatality rate was of only 9.6% (98). A woman suffering from both SARS-CoV-2 and CCHF was treated against both infections and she was lucky enough to beat both viral variants (99).

## 4. The CCHF virus and its association with humans

The life cycle of the CCHF virus depends upon a vector (ticks) and an amplifying host. There are many species of ticks that are proven to harbor the CCHF virus. The *Ixodes* genus of ticks is the most efficient among all other genera (100), while the *Hyalomma marginatum* is the most efficient vector (101). Other tick species that are reported to transmit the CCHF virus are *H. aegyptium, H. schulzei, H. onatoli, H. dromedarii, H. rufipes, H. excavatum, H. anatolicum, R. sanguineus, R. turanicus, R. annulatus, Ha. punctata, A. variegatum, H. truncatum, H. turanicum, I. ricinus, A. lepidum*, and *H. impeltatum* (74). *Hyalomma marginatum* has certain characteristics that make it a triumphant vector over others. It harbors the virus in its saliva and when the virus reproduces sufficiently in the intestinal tract of ticks then it replicates significantly and tends to spread to the other organs (102). Some organs have a low titer of the virus and few other organs possess a greater number of populations of the virus-like salivary gland and reproductive tract (103). The virus transmits vertically in different stages of life, other than adult stages, i.e., larvae and nymph (104). Female ticks have a tendency to lay thousands of eggs and the transovarian route is also present (105), so even a very low viral titer in females transfers to offspring and also circulates in the environment. These infected ticks also infect the non-infected animals in the vicinity, on the other hand, the infected animals also spread the CCHF virus to ticks that feed blood from infected animals (27). True natural CCHF reservoirs are those ticks that remain infective for a lifetime and never become free from the virus. The *Argasid* genus is not able to transfer virus vertically or horizontally (106).

**Figure 3:**
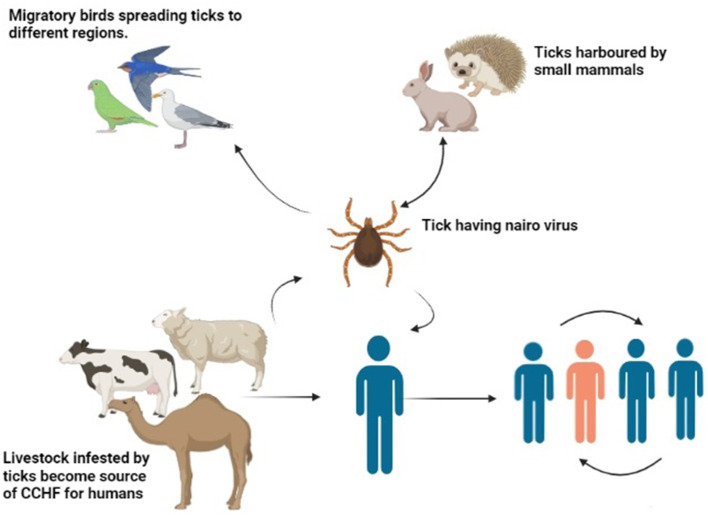
Transmission of CCHF virus (Drawn by BioRender app).

An amplifying host in the case of the CCHF virus is a vertebrate host, which amplifies the virus and transfers it to humans (107). The CCHF virus tends to multiply at a faster rate in blood and develops a high viral titer within or <14 days. Clinical symptoms are not observed in animals. Large herbivore animals are reported to be seropositive (108). Ostriches are the only birds that harbor the virus, and no other bird species has yet been reported to harbor the virus (109). Birds have a role in outspreading the disease during migration from one area to another as they carry infected ticks in their fur (110). Some small vertebrates, such as hares and hedgehogs, act as a reservoir, maintain the viral load in society, and tend to spread the disease, as illustrated in Figure 3 (111). They are usually infested with larval stages of ticks that transfer the virus in their life stages by vertical mode. On the other hand, large animals are infested with a great number of ticks at the same time (112). The condition can be worsened by the additive effect of horizontal transmission. Favorable conditions such as the hot and arid season help in the molting of tick larvae into the adult tick. Vegetation and humidity also help in the propagation of the ticks. Furthermore, the presence of large and small mammals in a particular area helps in the maintenance of the disease in a particular region. Researchers claim that the CCHF may spread to unaffected regions and countries of the Mediterranean region because they have a feasible environment for ticks (78). Some authors claim that in a few years, Western Europe will be facing a devastating outbreak of the disease because of its climatic adaptation and consistent distribution pattern of *Hyalomma* ticks (113).

## 5. Transmission and zoonotic impact of CCHF

Humans usually get bitten by an infected tick and develop the infection when they are rearing livestock and handling animals,. In some cases, humans remove ticks from animals and squash them by bare hand, ultimately developing the disease. Tick bites are responsible for 60 to 69% of patients (114). Adult ticks of the *Hyalomma* species complete their lifecycle in spring and summer whenthey feed on amplifying hosts (115). If the accompanied winter was not too harsh, the chances of CCHF cases increase as the tick population does not reduce (116). The ecosystem is also responsible for the spread of the disease in particular areas where a higher number of mammals, large or small, roam, with chances of a spillover to the human population reducing significantly because the virus tends to roam silently in those mammals, causing only sporadic cases of the disease in humans. On the other hand, in 1944 in the era of the Germans, livestock and farm animals were reduced significantly in Crimea, but the cases of CCHF in humans increased (27). Wild hares harbored the ticks rather than livestock and these hares transmitted infections to humans by the bite of *Hyalomma* ticks (27).

Infected tissues and blood of infected animals can also be the source of infection in the human population. An extensive study was done in Turkey from 2002 to 2007 that claimed that out of 1,820 patients, only 62% were in close association with animals (117). After the death of an animal, acidification of the internal environment of the body tends to reduce the viral load, but certain reports showed that tissue and blood infected with the virus can be problematic (108). Only 90% of cases were reported from patients that were professionally in contact with animals or animal tissue, such as farmers, butchers, and people working in slaughterhouses. A serological study revealed that older people have a high titer of anti-CCHF IgG antibodies and so do people who are in close contact with livestock or do not have a good socio-economic status. These people have a higher tendency to be bitten by ticks while handling livestock (118). Most human cases are observed in men because they are more likely to handle livestock and, in Middle Eastern countries where men are mostly performing outdoor jobs, are professionally adapted to animal-associated environments. Sexual transmission has been reported, but there are few such cases, so it has not been prioritized enough (119). In some cases, the virus was transferred vertically and the child showed symptoms of severe hemorrhage and ultimately died (120). On the other hand, infected mothers gave birth to non-infected babies, which suggests that vertical transmission from mother to child does not going happen in all cases of infected mothers (121).

Health workers usually get infected by accidental needlestick injuries while dealing with CCHF-positive patients. In Turkey, nine sites, where 4,869 patients of CCHF were admitted, were examined to check for the prevalence of the disease in health-care staff. Accidental exposure to CCHF was observed in 51 cases, and 25 of those developed CCHF. Among these accidentally exposed medical staff, 16% died due to the exposure. Among these 16%, needlestick injury-associated deaths were about 62.7%. Transmission due to body fluids was about 23.5% (115). Ribavirin was recommended to health workers who were accidentally exposed and, out of 32 patients, 19 were given prophylactic ribavirin and did not develop the disease. On the other hand, eight out of 13 developed clinical diseases and did not receive prophylactic treatment (122). On these bases, ribavirin is recommended as prophylaxis, but in this regard, no document has been formulated yet (122). The hemorrhagic phase of the disease is the riskiest in terms of transmission of the disease to health workers. No case has been reported during the incubation period of the disease (108). The absence of protective measures and the occurrence of needlestick injuries make health workers prone to the disease (108).

## 6. Pathogenesis of the disease

**Figure 4:**
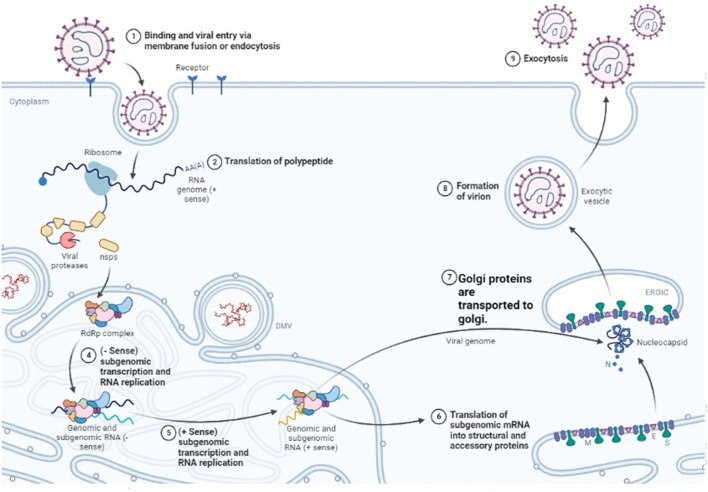
Replication cycle of Nairo virus within a host cell (Drawn by BioRender app).

The exact mechanism by which viruses produce pathogenic effects is not fully known. All the viruses that cause hemorrhagic fever have a common characteristic in which they disable the host's immune function and make it prone to the disease (123). They do so by attacking the antiviral cells. The virus starts to replicate speedily, as shown in Figure 4, and alters the normal functioning of the vascular system and lymphatic organs (124). CCHF pathogenesis is mainly dependent on the infection associated with the epithelium (125). The epithelium is damaged by the continuous replication of viral particles. The second method is indirect damage by the virus, in which the virus releases tissue-toxic factors or produces host-derived soluble factors that result in endothelial activation and loss of proper cellular functions. Damaged endothelium attracts the platelets to aggregate, and the intrinsic pathway of coagulation is activated. It is an early symptom that is obvious and ends as a hemostatic failure.

Cytopenia was associated with haemophagocytosis because it was a consistent finding in half of the patients in Turkey (93). High levels of Type 1 T helper cytokine, such as Tumor Necrosis Factor α, Interferon γ, Interleukin 6, and Interleukin 1, give rise to increased activation of monocytes that lead to haemophagocytic lymphohistiocytosis (126). In this study, it was also revealed that cytokines have roles in the pathogenesis of CCHF (93). It was further confirmed by another study that the level of Type 1 T helper cell cytokines was detected in patients who died and in those who survived. It was noted that the level of all these cytokines was lower in the patients who survived and higher in those who died because of that infection. In grave cases, Interleukin 6 and Tumor Necrosis Factor α levels were higher, along with disseminated intravascular coagulation. While Interleukin10 level was inversely related to them (127).

It was noted that cellular pathology was usually associated with the viral division among the cell, but the receptor that allows the virus to move into the cell was not identified yet. Protein domains that are present outside the cell, i.e., GC and GN glycoproteins, play an important role in the binding of the virus to the host cell. The nucleolin present in the host cell also plays a vital role in permitting the virus to cause cellular injury (128). Clathrin-dependent endocytosis enables the virus to enter the host cell (129). Upon entry into the cell, positive strand intermediates are made by viral RNA-dependent RNA polymerase (RdRp) when it interacts with encapsulated genome segments present in the cytoplasm of the host cell. With the help of these positive strands, their complementary negative strands are prepared. A model is drawn in which primer-independent manufacturing of both positive and negative sense genomes is claimed (130).

Microtubules of the host are the main players on which viral internalization, assembly, and egression are dependent (131). PreGN and PreGC, which are immature forms of GN and GC, are synthesized in the rough endoplasmic reticulum and upon their production, the synthesis of viral surface glycoprotein initiates. Both PreGC and PreGn are transferred to the Golgi body as a heterodimer. They are cleaved further, then the process of glycosylation occurs, and the heterodimer is folded and converted into a viral membrane (132). The Golgi body releases mature viruses into the neighboring environment, and this process is known as budding. In this way, the virus replicates and causes different degenerative changes.

## 7. Pathophysiology of the disease

Humans are the only known host that show clinical symptoms associated with the disease (133). According to a study, the chances of the development of the clinical disease in people harboring the virus were 0.215 to 1 among every five infected people (134). The development of the disease has four phases, which include an incubatory phase, in which replication of the virus happens in the body, the pre-hemorrhagic phase, the hemorrhagic phase, and the convalescent phase (135). The incubation period starts right after the infected tick bite and usually lasts between 3 to 7 days (136). The incubation period depends upon the amount of viral load injected during the bite and the route of exposure (137). Incubation period is shorter when tick directly feeds on blood rather than other transmission routes. Blood and tissue of infected animals take ~5 days to develop the infection. The human-to-human transmission also takes 5 to 7 days on average (138). The mean duration before presenting patients to the hospital is reported to be 3–5 days in UAE and 5 to 6 days in Turkey (13).

The second phase of the infection is the pre-hemorrhagic phase, in which the person with the infection shows signs of a fever that ranges from 39 to 41°C (139). There is severe headache, dizziness, and muscular pain (140). The patient remains with the fever for 4 to 5 days and then the fever subsides (63). In some cases, additional symptoms, such as diarrhea, vomiting, and nausea, are observed (141). This phase lasts for about 3 days and different parts of the body, such as face and neck, become hyperemic (142). The sclera becomes congested and conjunctivitis is usually observed (143).

The third phase of CCHF is the hemorrhagic phase, which is shorter and tends to be more prominent in terms of clinical symptoms because of hemorrhages. It usually appears on the 3rd to 5th day of the disease (144). No association is generally observed between fever and hemorrhages in patients (133). The shape of hemorrhages ranges from smaller ecchymotic to petechial hemorrhages. Large hematomas are present on the skin and the mucous membranes (145). Clotting time increases in patients suffering from CCHF and a stage comes when blood is thin enough to ooze out of natural orifices, such as the vagina, gingival tissues, and nose (15). Blood is also seen in urine (hematuria) and feces (melena), and bloody discharge also occurs from the uterus (Menometorrhagia) (146). Hemoptysis is also observed in the hemorrhagic phase (147). This phase is often confused with appendicitis if there is only internal bleeding and there is no sign of external bleeding (148). Persistent pain was thought to be caused due to inflammation in the appendix, but with further investigation, it was claimed that there were internal hemorrhages and bleeding in the cecum, and internal and external oblique muscle with no pathology related to the appendix (149). Hepatomegaly and splenomegaly were also observed in some patients suffering from CCHF, but it was not a consistent finding (150). These were the clinical features of CCHF patients who either recovered from that phase or died due to extensive bleeding and hemorrhages (151).

The last phase is the convalescent phase for those who survived the infection, and it starts about 10 to 20 days after the infection (152). Patients recovering from CCHF have a weak pulse, often accompanied by tachycardia, partial or complete alopecia, dyspnea, polyneuritis, xerostomia, deafness, memory loss, blindness, or weak eyesight (32). Some patients may have bradycardia and a drop in blood pressure (153).

## 8. Lab investigations in CCHF patients

**Figure 5:**
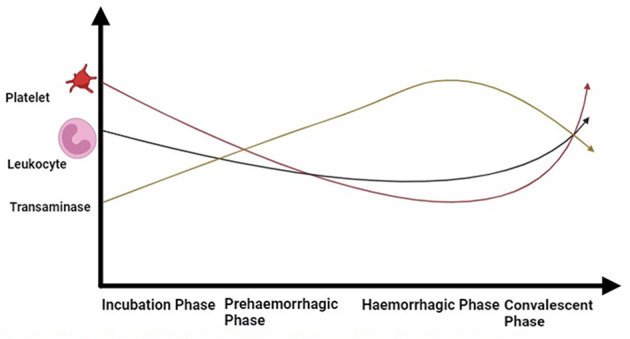
Dynamic of blood cells during different phases of CCHF in patients (Drawn by BioRender app).

The basic marker in the diagnosis of CCHF lab reports is a decreased level of platelets and leukocytes (Figure 5). Enzymes, such as aspartate aminotransferase, alanine aminotransaminase, creatinine phosphokinase, and lactate dehydrogenase, tend to increase. Prolonged clotting time is checked with a prothrombin test and an activated partial thromboplastin test. Fibrinogen is reduced, which tends to make a meshwork to bind platelets and protein to make a clot. An increase in the degradation products of fibrin could be observed (154). Within 5 to 9 days, the surviving patients' lab results tend to become normal.

## 9. Treatment

The treatment strategy of CCHF involves two aspects, one is to give symptomatic treatment to cover up the deficiencies that occur because of the extensive loss of blood cells, such as blood transfusion, platelets, or plasma is given to the patients (115). Hypovolemic patients are given electrolytes. Secondary infections are also addressed as there is immune suppression and the person becomes prone to other diseases (155). Any ongoing infection is also treated. For example, in some cases, malaria occurs along with CCHF in some patients (156).

Ribavirin is also used as treatment and, in some instances, it is given as prophylaxis. The WHO recommends the dose rate of ribavirin for patients. Firstly, it is recommended to give two grams of ribavirin per oral route. Then, until the 4th day, it should be given after an interval of 6 h and by a dose rate of 1 g. From day 5 to 10, it should be increased up to 500 g, with the same hour interval. Parental use has poor bioavailability, but it is used in some instances. The dose of ribavirin recommended is 17 mg/kg, which should not exceed 1 g until the 4th day and with an interval of 6 h. Then it is reduced to half (8 mg per kg) from day 5th to the 10th day. Ribavirin is considered safe with limited side effects and is used efficiently for the treatment of CCHF. Favipiravir, an antiviral drug, was tested in mice and reported to be better as compared to ribavirin, displaying results even when it was given to patients who exhibited symptoms of CCHF (157). However, due to a lack of enough evidence, it cannot be recommended for daily use for the treatment of CCHF.

## 10. Future perspectives

The exact manner of pathogenesis other than replication needs to be discovered. Research is needed to reveal the various mechanisms of disease production. Once identified, they can be used for the development of certain drugs or candidate vaccine virus that can block the pathway of development of infection by the virus. The impact of the enzootic environment needs to be examined in further detail. The transmission cycle of the CCHF virus and its vectors needs to be analyzed so that there may be a step from where we can break their cycle, ultimately resulting in the downregulation of the disease in a specific region. The field of pharmacology needs to excel to produce such antiviral drugs that can reduce the number of viruses in patients, either by killing the viruses or blocking their replication pathways. Death by CCHF is mostly attributed to disseminated intravascular coagulation. Anticoagulation factors, such as heparin and certain oxalates, can be tested to prevent Disseminated Intravascular Coagulation in patients and clinical symptoms of the disease. The mechanism by which the virus develops Disseminated Intravascular Coagulation in patients needs to be studied, and further studies on CCHF will result in the discovery of thisexact mechanism and how bacterial sepsis develops along with Disseminated Intravascular Coagulation. This understanding will lead to the development of drug molecules that will help to eliminate the disease around the globe. For this, all medical fields, including pharmacologists, pathologists, parasitologists, microbiologists, and clinicians have to work hand in hand to 1 day conquer the disease.

## 11. Conclusion

The presence of ticks and a suitable environment make CCHF an alarming disease in Asian and Middle Eastern countries. The prevalence of CCHF is noted to be increased per annum almost in all countries, including Pakistan, India, China, Iran, Kazakhstan, Egypt, Iraq, U.A.E, Saudi Arabia, and Turkey. Humans in close contact with livestock are at a greater risk than those in urban areas. Virus pathogenesis is attributed either by directly damaging cells by proliferation or indirectly by releasing cytotoxic compounds. Further investigations are required to discover the exact mechanism of the disease and to provide better healthcare to patients in different clinical phases of CCHF. To prevent zoonosis and transfer to medical health workers, certain measures should be taken to avoid the infection. Drugs with higher efficiency can be prepared once hidden mechanisms of disease are known. Prevention can be the key to success. Treatment can be undertaken using ribavirin and the medication can be given as prophylaxis. Vaccination development needs to be considered in the future for the advancement of better immunity in individuals.

## Author contributions

All authors listed have made a substantial, direct, and intellectual contribution to the work and approved it for publication.
